# Decoding Group Vocalizations: The Acoustic Energy Distribution of Chorus Howls Is Useful to Determine Wolf Reproduction

**DOI:** 10.1371/journal.pone.0153858

**Published:** 2016-05-04

**Authors:** Vicente Palacios, José Vicente López-Bao, Luis Llaneza, Carlos Fernández, Enrique Font

**Affiliations:** 1 Instituto Cavanilles de Biodiversidad y Biología Evolutiva, Universidad de Valencia, Valencia, Spain; 2 A.RE.NA. Asesores en Recursos Naturales, S.L. Lugo, Spain; 3 Research Unit of Biodiversity (UO/CSIC/PA), Universidad de Oviedo, Mieres, Spain; 4 Grimsö Wildlife Research Station, Swedish University of Agricultural Sciences (SLU), Riddarhyttan, Sweden; 5 Departamento de Matemáticas, Universidad de Oviedo, Oviedo, Spain; University of Windsor, CANADA

## Abstract

Population monitoring is crucial for wildlife management and conservation. In the last few decades, wildlife researchers have increasingly applied bioacoustics tools to obtain information on several essential ecological parameters, such as distribution and abundance. One such application involves wolves (*Canis lupus*). These canids respond to simulated howls by emitting group vocalizations known as chorus howls. These responses to simulated howls reveal the presence of wolf litters during the breeding period and are therefore often used to determine the status of wolf populations. However, the acoustic structure of chorus howls is complex and discriminating the presence of pups in a chorus is sometimes difficult, even for experienced observers. In this study, we evaluate the usefulness of analyses of the acoustic energy distribution in chorus howls to identify the presence of pups in a chorus. We analysed 110 Iberian wolf chorus howls with known pack composition and found that the acoustic energy distribution is concentrated at higher frequencies when there are pups vocalizing. We built predictive models using acoustic energy distribution features to determine the presence of pups in a chorus, concluding that the acoustic energy distribution in chorus howls can be used to determine the presence of wolf pups in a pack. The method we outline here is objective, accurate, easily implemented, and independent of the observer's experience. These advantages are especially relevant in the case of broad scale surveys or when many observers are involved. Furthermore, the analysis of the acoustic energy distribution can be implemented for monitoring other social canids that emit chorus howls such as jackals or coyotes, provides an easy way to obtain information on ecological parameters such as reproductive success, and could be useful to study other group vocalizations.

## Introduction

The integration of bioacoustics into wildlife management has grown extraordinarily in recent decades and provides efficient tools for monitoring several taxa such as amphibians [1], birds [2–5], or mammals [6–10]. The response of animals to sound playbacks has been used, for instance, to estimate population sizes and trends [11], which are essential population parameters to design and evaluate management and conservation actions [12].

Howls are key vocalizations in the intraspecific communication repertoires of several social canids and can be used as territorial displays, conveying information about pack location and minimizing contact between different packs [13–16]. Social canids such as coyotes (*Canis latrans)*, golden jackals (*C*. *aureus)*, and wolves (*C*. *lupus)* respond to human imitations of howls, which is the basis of the elicited-vocalization technique, a widely used method for detecting animals by their vocalizations [6,14,17]. In the case of wolves, this technique has been extensively used to estimate the number of wolf packs present in an area during the breeding period, since direct observation of wolves and wolf litters, especially in forests, is difficult and unsuitable for surveying large areas [6,17–22]. When several pack members respond to simulated howls they usually howl simultaneously, emitting what are known as chorus howls [6,23,24]. Chorus howls are complex acoustic signals. Besides howls, wolves can also emit other vocalizations in a chorus [15], including vocalizations described as part of the close-range vocal repertoire of wolves, such as barks, squeaks or growls [15,23–26], howl variations, such as 'woa-woa' howls [24,27], and vocalizations emitted only in group vocalization contexts, such as 'yips' [28].

From chorus howls emitted in response to simulated howls researchers obtain information regarding the presence of pups, which in turn is taken as evidence of the existence of a pack [21,29]. Surveys to confirm the presence of pups by means of howling are conducted in the summer and early autumn. At this time, pups remain at so-called “*rendezvous sites*” [30]. Two choruses emitted from locations several kilometres apart may be emitted by wolves belonging to the same pack. In these cases, the presence of pups howling and additional information obtained during surveys [29] allow researchers to estimate the existence of one or two different packs. Hence, obtaining reliable information regarding the presence of pups in a chorus is crucial to estimate the number of reproductive packs and to manage wolf populations efficiently.

Information regarding the presence of pups in a chorus can be obtained by means of: 1) acoustic assessment (i.e. listening to chorus howls), or 2) spectrographic analysis of recordings of chorus howls. Determination of the presence of pups by acoustic assessment is the most common method used for monitoring/management purposes [19–21,31,32] but, to date, no attempt to quantify the accuracy of such estimates has been made. Some researchers have used spectrograms to obtain information regarding group composition from chorus howls [19,20,22,33]. When pup presence is determined by spectrographic analysis, it is based mainly on the fundamental frequency of vocalizations, specifically howls, but no detailed descriptions of the methodology used for discriminating between adult and pup vocalizations have been published [19,20,22]. The use of fundamental frequencies to determine the presence of pups is sometimes a difficult task due to 1) the difficulty of identifying discrete vocalizations in an acoustic signal composed of multiple individuals emitting multiple vocalizations simultaneously, and 2) the scarcity of detailed descriptions of the repertoire of vocalizations that wolves emit in chorus howls. To our knowledge, to date no studies have addressed how factors such as pack size, sex, age and social status affect the structure of chorus howls. The few available descriptions of vocalizations included in chorus howls analysed only one vocal type (howls) [34] or different vocal types emitted only by adult wolves [24].

The spectral distribution of acoustic energy (energy transmitted by sound via propagating pressure fluctuations [35]) could provide a potentially useful alternative to the analysis of fundamental frequencies for detecting the presence of pups in chorus howls. Since pups emit vocalizations that are higher in pitch than those emitted by adults [36,37], we hypothesize that the acoustic energy of chorus howls with pups will be concentrated at higher frequencies than those emitted only by adult wolves. If this is so, analysing the acoustic energy distribution (hereafter AED) of a chorus howl should enable us to predict the presence of pups regardless of the different vocal types included in the chorus and their variability. The first aim of our study was therefore to explore the usefulness of the AED of chorus howls to obtain information regarding the presence of vocalizing wolf pups. Analyses of chorus howls have to take into account that choruses are long acoustic signals (up to several minutes), which include different types of vocalizations with distinct durations emitted by several individuals. Therefore, different parts of a chorus may have distinct acoustic structures that, in turn, affect the overall values of the AED and hence our ability to detect the presence of pups. In addition, analyses of recorded choruses have to consider the quality of the recording, differences in signal-to-noise ratio, and distortions due to the distance between the sender and the recording equipment. Thus, a second aim of our study was to determine the best procedure to predict the presence of pups using AED, taking into account 1) that chorus howls are long group vocalizations including different vocal types, and 2) differences in the quality of the recordings.

## Methods

### Data collection

Between 2000 and 2011 we recorded 110 chorus howls both in captivity (n = 74) and in the wild (n = 36). Most of the choruses (94.8%) were emitted in response to standardized human imitations of wolf howls [38], with only a few of them being emitted spontaneously. Human imitations of wolf howls are widely acknowledged as an appropriate method to elicit a vocal response from captive and wild wolves [6]. All the simulated howls were emitted by the same researcher (VP) and were standardized (no. of trials and pitch), thus minimizing the possibility of eliciting different types of responses to different stimuli [38,39]. Captive wolves were recorded with permission at four privately-owned nature preserves on the Iberian Peninsula: Cañada Real (Peralejos, Madrid, Spain), Carpín (Carranza, Bilbao, Spain), La Dehesa (Riopar, Albacete, Spain), and Centro de Recuperação do Lobo Ibérico (Malveira, Portugal). Wild wolves were recorded in five Spanish provinces: Lugo, A Coruña, Pontevedra (Galicia), Asturias, and Zamora (Castilla y León). Recordings of wild wolves were obtained during the course of local wolf monitoring programmes conducted in Galicia and Asturias, approved by the Dirección Xeral de Conservación da Natureza, Xunta de Galicia, and the Consejería de Medio Ambiente, Principado de Asturias, or were specifically approved by the Servicio Territorial de Medio Ambiente de Zamora, Junta de Castilla y León, following relevant national and international guidelines.

Most of the recordings (87%) were made on TDK SA-60 cassette tapes (TDK Electronics Corp., New York) using a Sennheiser MK 66 unidirectional microphone with a K-6 power unit (Sennheiser Electric GmbH & Co. kG, Wedemark, Germany) connected to a Marantz PMD 222 cassette recorder (Marantz America, Inc., Mahwah, New Jersey). These recordings were digitized with a 44.1 kHz sampling frequency and 16 bits in the Fonoteca Zoológica, Museo Nacional de Ciencias Naturales (CSIC, Madrid, Spain), using Delta 66 (Irwindale, California) or Digi 001 (Bucks, United Kingdom) digitizer cards. The remaining recordings were obtained using a Sennheiser MKH 70 directional microphone attached to a Marantz PMD 670 solid-state recorder. These recordings were saved as.WAV files with a 44.1 kHz sampling frequency and 16 bits.

### Data analyses

For each chorus, we generated spectrograms (2048-point fast Fourier transform; Hann window; bandpass filter 200–2500 Hz; frequency resolution: 21.5 Hz), measured the percentage of time the chorus was comprised exclusively of howls or of other vocalizations in addition to howls, and measured a total of 10 acoustic variables relating to AED (Table 1) using Raven Pro 1.4 (Cornell University Laboratory of Ornithology, www.birds.cornell.edu/raven). Furthermore, we used custom-made software to measure three additional variables: mean AED (AED-M), AED standard deviation (AED-SD), and peak AED (AED-P) (see description and procedures used to calculate these variables in Table 1 and S1 Appendix).

**Table 1:** Description of the acoustic features measured.

Variable	Description
**Q1Freq**	Frequency that divides the spectrum into two frequency intervals containing 25% and 75% of the energy (Hz)
**Q3Freq**	Frequency that divides the spectrum into two frequency intervals containing 75% and 25% of the energy (Hz)
**IQRBW**	Difference between the 1st and 3rd Quartile Frequencies. Q3Freq—Q1Freq (Hz)
**AggEntropy**	Aggregate entropy: measurement of the disorder in a sound by analysing the energy distribution within a selection. Higher entropy values correspond to greater disorder in the sound, whereas a pure tone with energy in only one frequency bin would have a value of zero [40]
**AvgEntropy**	Average entropy: average of the entropy for each frame in the selection [40]
**CentFreq**	Frequency that divides the spectrum into two frequency intervals of equal energy (Hz)
**Freq5**	Frequency that divides the spectrum into two frequency intervals containing 5% and 95% of the energy (Hz)
**Freq95**	Frequency that divides the spectrum into two frequency intervals containing 95% and 5% of the energy (Hz)
**BW90**	The difference between the 5% and 95% frequencies. Freq95—Freq5 (Hz)
**MaxFreq**	The frequency at which the maximum amplitude occurs (Hz)
**AED-M**^**1**^	Frequency corresponding to the mean energy density (Hz)
**AED-SD**^**1**^	Standard deviation of the mean energy density (Hz)
**AED-P**^**1**^	Frequency corresponding to the peak of the energy density (Hz)

AED: acoustic energy distribution (spectral distribution of the energy transmitted by sound via the propagating pressure fluctuations).

^1^: variables measured using custom made software (see S1 Appendix)

We classified choruses into two groups: with and without pups. For all choruses recorded in captivity, we knew the individuals participating in the chorus. However, most of the recordings obtained in the wild were made at night or in areas with scarce visibility. Choruses with pups were initially identified as those recorded between July and December, when newborn wolves are less than 6–7 months old, since from this age on the acoustic characteristics of the vocalizations emitted by young wolves coincide with those of the adult wolves [15,23]. Choruses recorded outside this period were considered to be produced by yearlings, subadults or adults. In four out of 36 recordings in the wild it was possible to confirm visually that there were pups vocalizing. In the remaining cases we considered that pups participated in the chorus if the following requirements were simultaneously met: i) visual confirmation of the presence of pups at the location where the recording was obtained within 12 h (before or after) of the moment the recording was made; and ii) the chorus included vocalizations consistent with published descriptions of pup vocalizations [36,37] and with the same acoustic structure as vocalizations of pups included in our own recordings of visually-confirmed pups. Thus, we included in the analyses 33 recordings classified as with pups: six with visual confirmation of pups vocalizing (two in captivity and four in the wild) and 27 that fitted the above criteria. We compared the acoustic features of choruses with visual confirmation of the presence of pups and those classified as with pups according to our criteria and found no statistically significant differences (Mann-Whitney U tests, Q1Freq: U = 75.5, p = 0.82; Q3Freq: U = 66, p = 0.5; IQRBW: U = 56.5, p = 0.26; AggEntropy: U = 62, p = 0.4; AvgEntropy: U = 117, p = 0.1; CentFreq: U = 80, p = 0.98; Freq5: U = 89, p = 0.73; Freq95: U = 74, p = 0.76; BW90: U = 74.5, p = 0.78; MaxFreq: U = 81, p = 1; AED-M: U = 75, p = 0.8; AED-SD: U = 71.5, p = 0.67; AED-P: U = 81, p = 1). Therefore, we assumed that all the choruses classified as with pups effectively included vocalizing pups.

**Fig 1:**
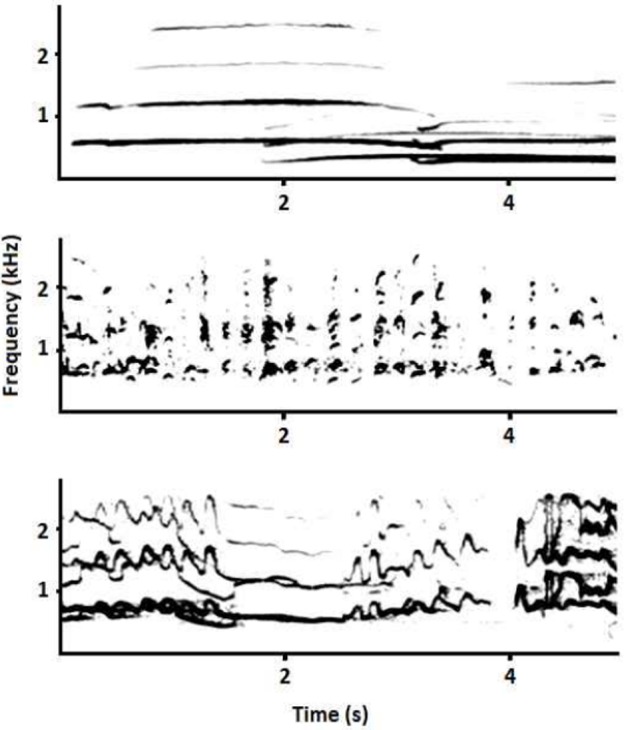
Fragments of chorus howls including different types of vocalizations. Fragments of chorus howl including howls (top), short vocalizations (middle), and long highly-modulated vocalizations (bottom).

Chorus howls are long [15] and include several vocalizations of different durations emitted by more than one individual. Because of this complexity, different parts of a chorus howl may have distinct acoustic structures in turn affecting the overall values of the AED and hence our ability to detect the presence of pups (Fig 1). We used two different approaches to analyse each chorus: 1) considering the entire duration of the chorus (CHORUS), and 2) segmenting/dividing the chorus into small 5 s fragments (SEGMENTS), since the longest vocalization is the howl and the majority of Iberian wolf howls are 4–8 s long [38]. In the first case, we measured the 13 acoustic variables mentioned above (Table 1) for the entire chorus, whereas when choruses were divided into segments we calculated mean, minimum, maximum and standard deviation for each of the same 13 acoustic features for all the segments comprising a chorus.

To reduce biases due to the distance of the sender from the microphone and different signal-to-noise ratios, we measured the acoustic features at two amplitude thresholds (75% and 90% peak amplitude) following recommendations made by Schrader and Hammerschmidt [41]. We therefore compared the effectiveness of four different approaches for determining the presence of pups using AED: entire choruses applying 75% (CHORUS75-model) and 90% (CHORUS90-model) amplitude thresholds, and choruses divided into segments with 75% and 90% amplitude thresholds (SEGMENTS75-model and SEGMENTS90-model respectively).

For each dataset, we conducted Mann-Whitney U tests to select those variables that showed significant differences (using Bonferroni's adjusted critical p-values to account for multiple tests) between choruses with and without pups. Then, in order to obtain the most parsimonious models determining the presence of pups in choruses, we used a two-step modelling approach. For each dataset (CHORUS75-model, CHORUS90-model, SEGMENTS75-model and SEGMENTS90-model), we first built univariate generalized linear mixed models (GLMMs) using only the significant variables from the Mann-Whitney U tests, with binomial error distribution and logit link, to test for differences between choruses with and without pups. Pack was treated as a random effect to avoid pseudoreplication due to the existence of various choruses from the same packs. We selected the variables that showed significant differences between choruses with and without pups in the GLMMs and removed those that were highly correlated (Pearson correlation coefficient ≥ 0.9) in order to avoid multicolinearity. In a second step, we built a set of competing GLMMs considering all the possible combinations using the selected variables in each dataset (including the null model, i.e. the intercept-only model). We used Akaike’s Information Criterion (AIC) to rank models, selecting the model with the lowest AIC values [42]. Cumulative AIC weights were calculated to evaluate the strength of each model. For each model, we estimated the marginal and the conditional R^2^ following Nakagawa and Schielzeth [43]. The best models were used to make predictions based on the original dataset to see the percentage of correct predictions and to analyse the type of errors produced by the models. All GLMMs were fitted in R software [44] using the *“lme4”* package [45].

## Results

We analysed 110 wolf chorus howls emitted by 20 packs, 77 comprising only adults and 33 that included pups (Table 2). Iberian wolf choruses are long vocalizations (26 to 183 s) with a mean (± SD) duration of 66 ± 27 s. AED was concentrated between 300 and 1900 Hz (minimum and maximum values of the variables Freq5 and Freq95 respectively). Howls were the main vocalization (more than 50% of the chorus duration) in 33% of cases, while the remaining 67% primarily included other vocalizations, such as highly modulated sounds, howl variations, and barks. The number of choruses composed mainly of howls was significantly greater for choruses emitted only by adults than those including pups (χ^*2*^ = 7.609; d.f. = 1; p = 0.006; Table 2).

**Table 2:** Choruses analysed in this study.

Age class	Pack	N	Main vocal types	
			Howls	Other
**Without pups (only adults)**	C1	11	43%	57%
	C2	2		
	C3	3		
	C4	5		
	CA	2		
	CR1	31		
	CR2	13		
	D1	3		
	D2	2		
	F	2		
	PO	1		
	S	1		
	T	1		
**With pups (adults and pups)**	A	1	21%	79%
	F	5		
	PE	1		
	PO	8		
	R	2		
	T	2		
	TI	1		
	TO	4		
**With pups (only pups)**	A	1	0%	100%
	C2	2		
	P	1		
	PI	1		
	PO	4		

N: number of choruses. Main vocal types refer to the vocalizations present in more than 50% of the entire length of the chorus. The code assigned to each pack corresponds to the initials of the pack's location.

The acoustic energy of chorus howls with pups was concentrated at higher frequencies and showed values of entropy that were higher than in choruses without pups (Table 3). The values of most of the variables measured (62%) were different in choruses with and without pups (Table 3). The best models considering the entire chorus (CHORUS-based models) were obtained for the frequency that divides the signal into two frequency bands with equal energy (CentFreq) and the frequency that divides the signal into two frequency bands containing 5% of the energy (Freq5) for the 75% amplitude threshold, and CentFreq for the 90% amplitude threshold (Table 4). The best SEGMENTS-based models were obtained for the mean value of Freq5, the maximum and mean values of Freq5, and mean average entropy (Table 4).

**Table 3:** Differences between the acoustic energy distribution parameters obtained for choruses with and without pups (Mann-Whitney U tests).

Variable	Without pups	With pups	U	p
	Mean ± SD	Mean ± SD		
**Q1Freq (Hz)**	520 ± 106	693 ± 139	411.5	< 0.001*
**Q3Freq (Hz)**	816 ± 249	1016 ± 237	684.5	< 0.001*
**IQRBW (Hz)**	296 ± 186	323 ± 158	1088.5	0.236
**AggEntropy**	4.83 ± 0.71	5.26 ± 0.44	841	0.005
**AvgEntropy**	2.91 ± 0.38	3.69 ± 0.44	253	< 0.001*
**CentFreq (Hz)**	629 ± 163	859 ± 189	420	< 0.001*
**Freq5 (Hz)**	417 ± 66	543 ± 98	363	< 0.001*
**Freq95 (Hz)**	1205 ± 345	1419 ± 268	864	0.008
**BW90 (Hz)**	789 ± 320	876 ± 266	1141	0.4
**MaxFreq (Hz)**	721 ± 290	955 ± 289	596.5	< 0.001*
**AED-M (Hz)**	872 ± 156	1036 ± 133	596.5	< 0.001*
**AED-SD (Hz)**	438 ± 65	450 ± 43	1218.5	0.737
**AED-P (Hz)**	574 ± 171	812 ± 288	488	< 0.001*

*: statistically significant, Bonferroni's adjusted critical p-value = 0.0038

**Table 4:** GLMMs obtained considering the different datasets.

CHORUS75-models	df	AICc	Delta	Weight	R^2^m	R^2^c
CentFreq, Freq5*	4	65.63	0.00	0.57	0.37	0.89
*CentFreq*	3	67.14	1.52	0.27		
**CHORUS90-models**	**df**	**AICc**	**Delta**	**Weight**	**R**^**2**^**m**	**R**^**2**^**c**
CentFreq*	3	68.62	0.00	0.47	0.09	0.87
*Q1Freq*	3	69.42	0.80	0.32		
*CentFreq*, *Q1Freq*	4	70.58	1.97	0.18		
**SEGMENTS75-models**	**df**	**AICc**	**Delta**	**Weight**	**R**^**2**^**m**	**R**^**2**^**c**
Mean-AvgEntropy, Mean-Freq5*	4	59.23	0.00	0.16	0.47	0.91
*Mean-AvgEntropy*, *Min-Freq5*	4	60.22	0.99	0.10		
*Mean-Freq5*	3	60.30	1.07	0.09		
*Mean-AvgEntropy*, *Mean-Freq5*, *Min-Freq5*	5	60.31	1.08	0.09		
*Max-AvgEntropy*, *Mean-Freq5*	4	60.89	1.66	0.07		
*Mean-AvgEntropy*, *Mean-Freq5*, *Min-AED-P*	5	61.15	1.92	0.06		
**SEGMENTS90-models**	**df**	**AICc**	**Delta**	**Weight**	**R**^**2**^**m**	**R**^**2**^**c**
Max-Freq5, Mean-AvgEntropy, Mean-Freq5*	5	56.68	0.00	0.16	0.87	0.92
*Max-Freq5*, *Mean-Freq5*	4	58.08	1.40	0.08		
*Max-Freq5*, *Mean-AvgEntropy*, *Mean-Freq5*, *Min-Q1Freq*	6	58.18	1.50	0.07		
*Mean-AvgEntropy*, *Mean-Freq5*	4	58.53	1.86	0.06		
*Max-Freq5*, *MaxQ1Freq*, *Mean-AvgEntropy*, *Mean-Freq5*	6	58.57	1.89	0.06		

*: Best models considering the AIC criterion for each dataset; df: number of parameters in the model; AICc: Akaike’s information criterion; Delta: Delta AIC value; Weight: Akaike weight; R^2^m: marginal R^2^; R^2^c: conditional R^2^. For the sake of simplicity, only models within ∆AICc< 2 are shown.

The best values of R^2^ were obtained for the SEGMENTS90-model, explaining 92% of the variance, only 5% due to random effects (Table 4). The accuracy of the predictions was better for the analyses performed dividing the choruses into segments (e.g. the SEGMENTS90-model predicted the presence of pups in 84.8% of choruses with pups while this percentage decreased to 27.3% with the CHORUS90-model, Table 5). The best results were obtained for the SEGMENTS90-model: in 93.6% of choruses used to build the models this model correctly predicted the presence of pups. All the models had low percentages of false positives: only in 3.9–6.5% of cases, when no pups vocalized, did models wrongly predict that pups were present (Table 5).

**Table 5:** Correct and wrong predictions on applying the best models.

Model	Correct predictions	Presence of pups	Model prediction	
			0	1
**CHORUS75**	81.8%	0	96.1%	3.9%
		1	51.5%	48.5%
**CHORUS90**	73.6%	0	93.5%	6.5%
		1	72.7%	27.3%
**SEGMENTS75**	85.5%	0	96.1%	3.9%
		1	39.4%	60.6%
**SEGMENTS90**	93.6%	0	97.4%	2.6%
		1	15.2%	84.8%

We considered that a chorus howl included pups when the probability of pups vocalizing on applying the model > 0.5.

## Discussion

We studied the acoustic energy distribution (AED) of chorus howls emitted by Iberian wolves to investigate whether the acoustic properties of such choruses could encode information regarding the presence of vocalizing pups. The acoustic energy of chorus howls was concentrated at lower frequencies when there were only adults vocalizing. Most of the vocalizations included in chorus howls are harmonic sounds and the harmonic signals emitted by terrestrial carnivores undergo a decrease in fundamental frequency, upper frequency limit, frequency range, and harmonic with highest intensity with increasing age [46]. It has been reported that some wolf vocalizations show age-related changes in the fundamental frequencies, which probably reflects, at least in part, the growth of the vocal tract [47]. For instance, the fundamental frequency of howls drops from an average of about 1100 Hz at two weeks [37] to about 350 Hz by 6–7 months of age [34,36]. In our study, the frequency that divides the signal into two bands containing 50% of the acoustic energy increased from an average of 600 Hz in choruses without pups to 850 Hz when there were pups vocalizing.

Besides frequency variables, entropy values of choruses with pups were higher than those of choruses without pups. Entropy has been used to study other acoustic signals, for instance to estimate call linearity of choruses emitted by southern pied babblers [48], and it has also been used as an acoustic index to monitor animal diversity [49]. Vocalizations emitted by wolves in a chorus that were highly modulated and noisy yielded high entropy values, whereas pure tones had lower entropy [40]. We found that chorus howls with pups vocalizing included a greater proportion of vocalizations other than howls, which could explain the results obtained.

Our results suggest that, despite their complexity, chorus howls can be used to determine quantitatively, objectively and with a high degree of certainty, that a pack of wolves contains pups and has therefore reproduced successfully. We built different models to predict the presence of pups in a chorus howl based on the analysis of the AED. Each approach has its advantages and disadvantages. SEGMENTS-based models, for example, have more predictive power than CHORUS-based models. The major differences between CHORUS and SEGMENTS models have to do with the percentage of true positives and false negatives (Table 5). When there are pups vocalizing, the CHORUS75-model detects their presence (true positive) 49% of the time, whereas this percentage increases up to 85% with the SEGMENTS90-model. False negatives (pups are present but the probability of there being pups according to the model is < 0.5) are lower for SEGMENTS-based models. With the SEGMENTS-based models, a 90% amplitude threshold yields more accurate results. The application of both CHORUS- and SEGMENTS-based models provides low percentages of false positives (the model predicts that there are pups when there are no pups vocalizing, Table 5). In addition, CHORUS-based models are less time-consuming than SEGMENTS-based models. We estimate that the analysis of a chorus with a CHORUS-based model can be completed in 30 min, whereas the same chorus analysed with a SEGMENTS-based model would require between 2 and 3 h. Therefore, CHORUS-based models provide less accurate results and underestimate the presence of pups, but are less time-consuming than SEGMENTS-based models. However, whenever possible, we recommend the use of the SEGMENTS90-model. We are aware that the inclusion of choruses without visually-confirmed pups vocalizing may compromise the interpretation of our results. The absence of significant differences between choruses with and without visual confirmation of pups may be the result of an insufficient sample size and does not necessarily imply that no differences exist. However, all choruses with pups included to build the models were chorus howls emitted during the breeding season at *rendezvous sites* by packs of wolves that reproduced successfully. Hence, it seems safe to conclude that the models can indeed be used to estimate the presence of pups in a pack of wolves. Nevertheless, further research is needed to clarify this issue.

The elicited-vocalization technique has proved to be useful for estimating the number of wolf packs [6,21,29]. The present results show that this technique, when combined with analyses of chorus howl recordings, can also be used to confirm reproduction in a pack. Spectrographic analyses based on the analysis of the AED in chorus howls can be used to determine, with a high degree of accuracy, that a pack of wolves contains pups. Other methods have been proposed to determine the presence of pups in a chorus, e.g. acoustic assessment or analyses of the fundamental frequencies of the vocalizations, but no attempt to assess their reliability has been made [19–22,31,32]. Particularly when the aim is to survey wide areas, AED predictive models present important advantages with respect to other approaches: 1) this method does not require an in-depth knowledge of the repertoire of vocalizations included in chorus howls, and 2) predictions are objective and repeatable, and the results do not depend on the experience of the observer. Furthermore, the probability of incorrect predictions and the type of error has to be taken into account when management decisions pertain to endangered or harvested populations. To wrongly claim the presence of pups when there are in fact none (false positives) is possibly the most undesirable mistake, although it can be minimized by applying predictive models (2.6–3.9% using predictive AED models).

Simulated howling is one of the most commonly used methods to monitor wolf populations [6,20,21,29,50,51]. In Europe, for instance, this method is systematically used in Finland, Estonia, Romania, Bosnia-Herzegovina, Bulgaria, Greece, Slovenia, Italy, France, Portugal and Spain [52]. What we propose is not a new field technique but rather the application of predictive models to chorus recordings in order to confirm the presence of pups in a pack. Recording chorus howls increases the costs of monitoring programmes, but modern recording devices are small, portable and relatively cheap. When the aim of the survey is to estimate reproductive rates or population trends, or when management decisions depend on the results of the survey, it is important to apply the most reliable and accurate methodology [12]. Therefore, the costs associated to equipment acquisition are offset by the benefits of applying predictive models that are objective, accurate and repeatable.

The methodology proposed here could be extended, for instance, to monitor other species emitting similar group vocalizations, such as other social canids (e.g. jackals or coyotes [14,17,53]). In addition, the analysis of AED provides an easy way to obtain information from group vocalizations (multiple vocalizations emitted by several individuals simultaneously) without analysing or isolating single vocalizations. The acoustic structure of these single vocalizations may vary with sex, age, context or status, which could affect the AED of the overall signal (e.g. [48]). AED could thus be used to analyse group vocalizations or chorus vocalizations emitted by other animals, for instance, to test whether individuals of a specific sex or status are present, thereby providing information about group composition. The potential of AED to monitor wildlife populations and study other acoustic signals used in animal communication deserves further investigation.

## Supporting Information

S1 Appendix Appendix I. (DOCX)
