# Supplementary material for: Decoding Group Vocalizations: The Acoustic Energy Distribution of Chorus Howls Is Useful to Determine Wolf Reproduction
Source: PLoS One. 2016 May 4;11(5):e0153858. doi: 10.1371/journal.pone.0153858 (PMC4856277; doi:10.1371/journal.pone.0153858)
Supplement: S1 Appendix — (DOCX) [file pone.0153858.s001.docx]

**APPENDIX I**

We used custom made software to measure three variables related to the AED: AED mean (AED-M); AED standard deviation (AED-SD), and AED peak (AED-P). The procedure to calculate these variables follows the next steps:

1) We calculate the Fourier transform

$$\left( \Gamma_{v}f \right)\left( \tau,\omega\right)= \int_{-\infty}^{\infty} f\left( t \right)v\left( t-\tau\right)\exp\left[ -it\omega\right]dt$$

where τ is time and ω is frequency.

2) The function

$$ℇ\left( \tau,\omega\right)= \left| \left( \Gamma_{v}f \right)(\tau,\omega) \right|^{2}$$

yields the energy of the signal at time τ and frequency ω

3) In this context, the integral

$$\left\| f \right\|^{2}= \frac{1}{2\pi}\iint_{\mathbb{R}^{2}} ℇ\left( \tau,\omega\right)d\omega d\tau$$

corresponds to the total energy of the signal.

4) We calculate the average energy of the signal in the time

$$\left( E_{\tau} \right)\left( \omega\right)= \frac{1}{\left\| f \right\|^{2}}\int_{-\infty}^{\infty} \tauℇ\left( \tau,\omega\right)d\tau$$

This function only depends on the frequency $\left( E_{\tau}f \right)\left( \omega\right)$ and yields the percentage of energy corresponding to frequency ω

5) Then we calculate the mean value of the frequency energy density

$\mu= E\left[ \left( E_{\tau}f \right) \right]= \frac{1}{2\pi}\int_{-\infty}^{\infty} \omega\left( E_{\tau}f \right)\left( \omega\right)d\omega$,

and its standard deviation

$\sigma^{2}= V\left[ \left( E_{\tau}f \right) \right]= \frac{1}{2\pi}\int_{-\infty}^{\infty} \left( \omega-\mu\right)^{2}\left( E_{\tau}f \right)\left( \omega\right)d\omega$,

6) AED-P is the frequency corresponding to the maximum energy density.
